# Supplementary material for: Opposite effects of positive and negative symptoms on resting-state brain networks in schizophrenia
Source: Commun Biol. 2023 Mar 17;6:279. doi: 10.1038/s42003-023-04637-0 (PMC10023794; doi:10.1038/s42003-023-04637-0)
Supplement: Supplementary file 2 — Supplementary Information [file 42003_2023_4637_MOESM2_ESM.pdf]

Supplementary Information

Opposite effects of positive and negative symptoms on resting-state brain networks in schizophrenia

Xinrui Wang[1#], Zhao Chang[1#], Rong Wang[1]*

[1]College of Science, Xi'an University of Science and Technology, Xi'an, Shaanxi, China

# These authors contributed equally.

* Corresponding author: wang0712_xust@163.com (RW).

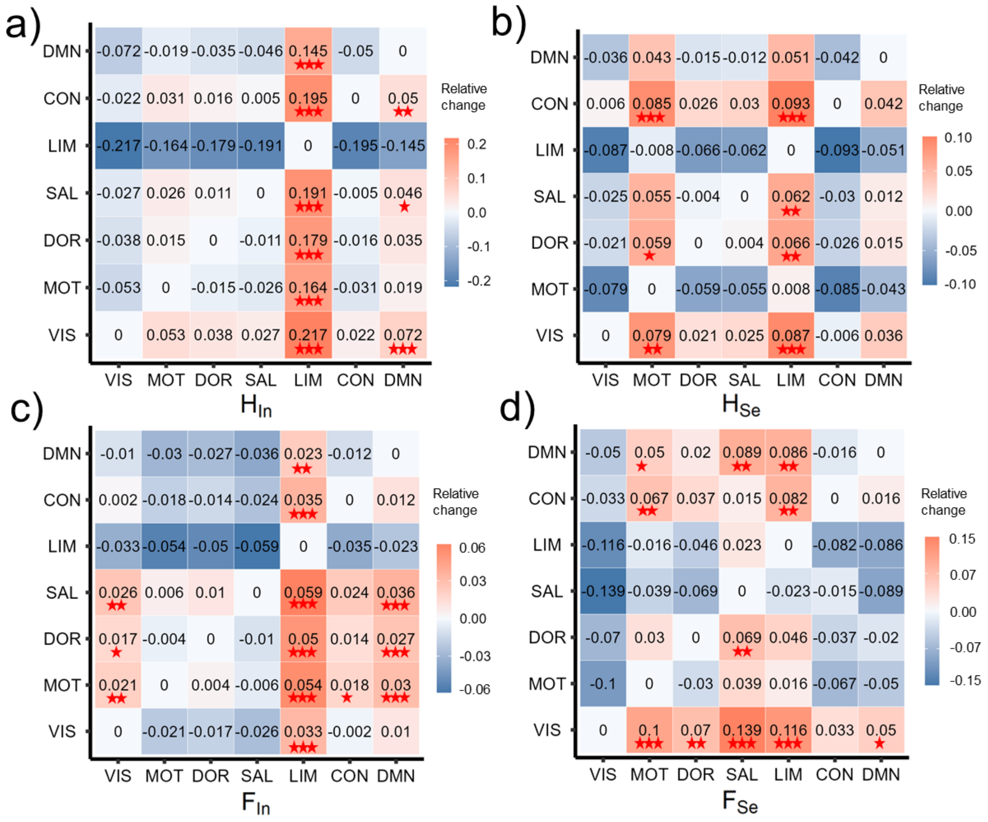

Supplementary Figure 1. Permutation tests for relative changes of systems from HC to SCH. The relative change of each system was calculated as the $S = (S_{SCH} - S_{HC}) / S_{HC}$ wherein S_{SCH} and S_{HC} indicate one of brain measures in SCH and HC groups. The S for the regions in each system was averaged to represent the relative change of this system. Then, the difference of relative change between different systems was calculated. In the difference matrix, each element (i, j) indicates the difference between system *j* and systems *i*, e.g, the column of LIM means that the relative change in LIM minus that in other systems. We performed the permutation test to test the difference. *S* was randomized across regions 1000 times, and the corresponding permutated *S* was obtained for each region. Then, the difference of *S* between different systems was calculated, and the corresponding probability (*p*-value) was obtained, ****p*<0.001, ***p*<0.01, **p*<0.05. The limbic system had the largest alterations in brain measures.

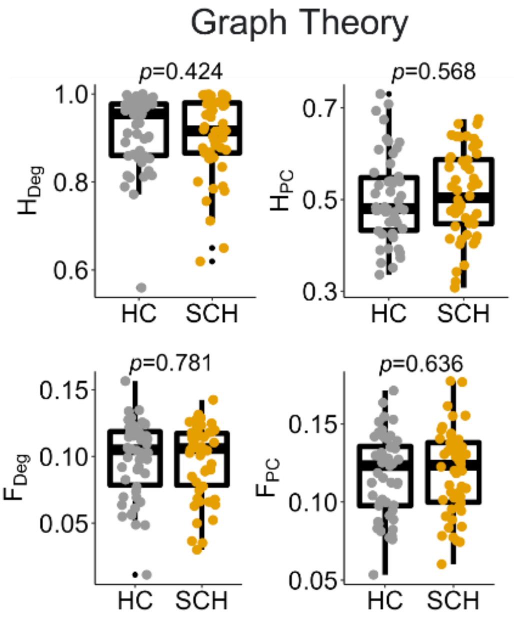

Supplementary Figure 2. Brain network measures for graph theory. We calculated the corresponding graph theory measures: degree, participant coefficient, variability of degree and variability of participant coefficient. There are no significant differences in graph theory measures between HC and SCH groups, but our NSP method can detect the differences (Fig. 1).

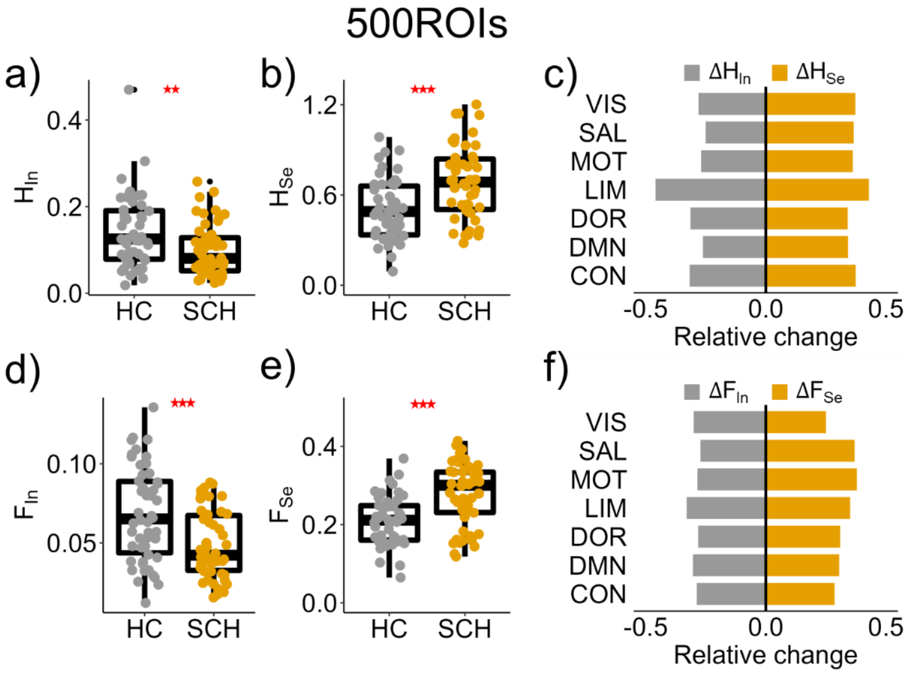

Supplementary Figure 3. Dynamic segregation and integration for 500 brain regions. a) We calculated the results for the brain parcellation of 500 regions using the Schaefer atlas. Compared to the HC group, the SCH group had decreased integration strength ($t(98)=-2.670$, $p=0.004$) and **b)** increased segregation strength ($t(98)=3.766$, $p<0.001$). **c)** All functional systems had significantly decreased integration strength and increased segregation strength, and the limbic system had the largest alterations. **d)** The SCH group also had decreased integration variability ($t(98)=-3.623$, $p<0.001$) and **e)** increased segregation variability ($t(98)=5.253$, $p<0.001$). **f)** The limbic system had high decrease in integration and segregation variabilities. These results are similar to those from the parcellation of 200 regions. VIS-visual; MOT-motor; DOR-dorsal attention; SAL-salient attention; LIM-limbic; CON-control; DMN-default-mode network.

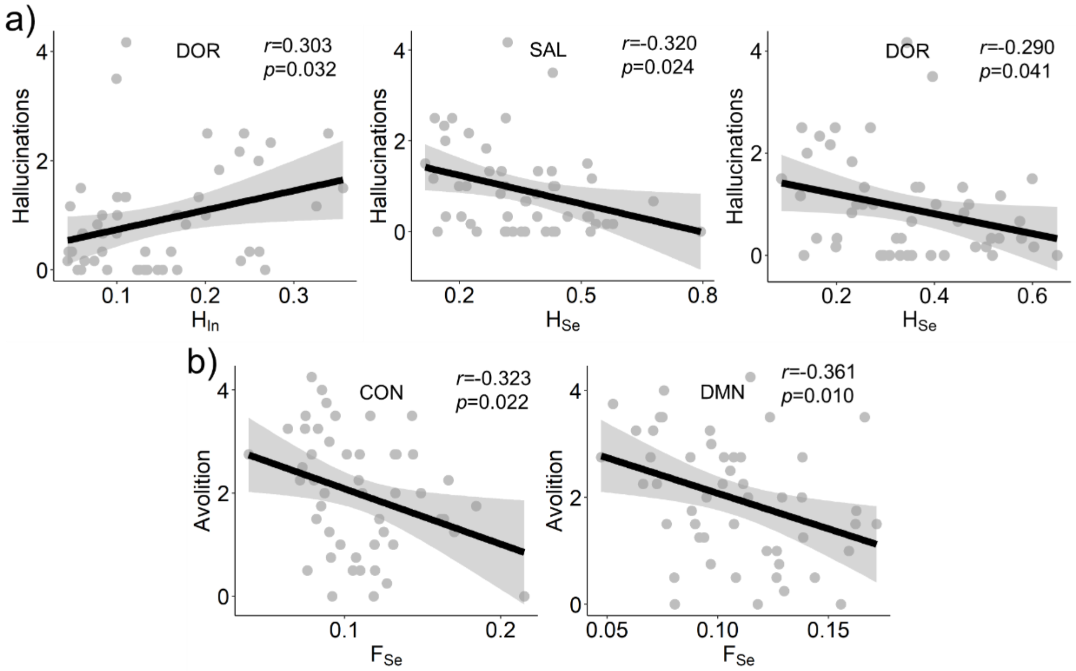

Supplementary Figure 4. Correlations between schizophrenia symptoms and brain network measures for 200 regions. a) Hallucinations symptom is predicable from integration strength H_{In} of the dorsal attention system and segregation strength H_{Se} of salient attention and dorsal attention systems. **b)** Avolition symptom is related to network segregation variability F_{Se} of control and DMN systems.

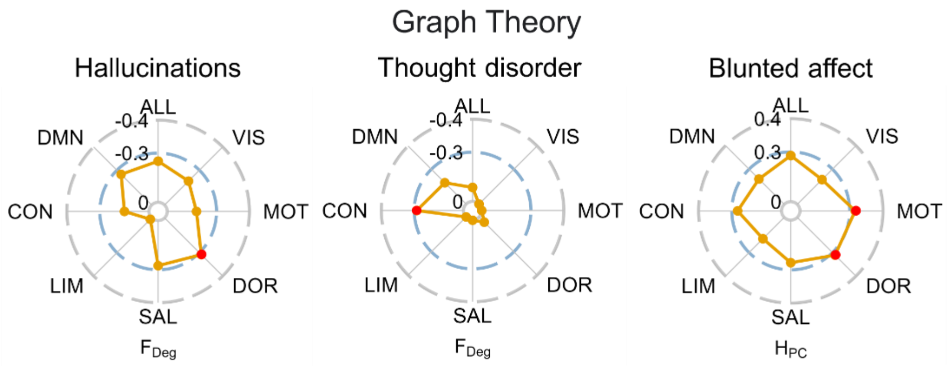

Supplementary Figure 5. Significant correlations of symptom scores to graph theory measures for the whole-brain (ALL) and seven functional systems. The dynamic degree variability F_{Deg} was negatively related to hallucinations score in the dorsal attention system (*r*=0.316, *p*=0.025) and thought disorder score in the control systems (*r*=-0.285, *p*=0.045). The blunted affect score is positively correlated with the participation coefficient strength H_{PC} in the dorsal attention system (*r*=0.322, *p*=0.023) and motor system (*r*=0.333, *p*=0.018). Graph theory and NSP measures have consistent correlations to key positive symptoms (i.e., hallucinations); graph theory measures are also related to positive symptoms thought disorder and blunted affect, but only NSP measures are related to negative symptoms (i.e., avolition).

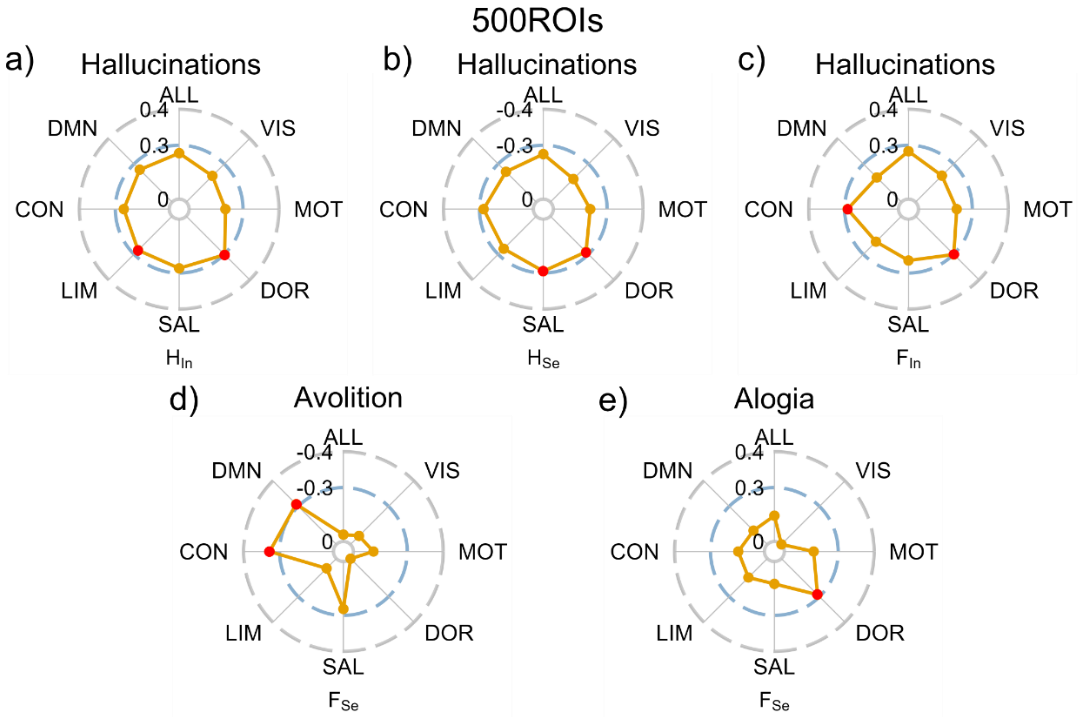

Supplementary Figure 6. Relationships between schizophrenia symptoms and brain networks with 500 regions. a) The hallucinations are positively correlated with integration strength H_{In} in the dorsal attention system (r=0.290, p=0.041) and limbic system (r=0.292, p=0.040), **b)** negatively related to the segregation strength H_{Se} in the salient attention system (r=-0.290, p=0.041) and the dorsal attention system (r=-0.281, p=0.048). **c)** The variability of the dynamic integrating process was positively related to the hallucinations score in the dorsal attention system (r=0.302, p=0.033) and control systems (r=0.284, p=0.046). **d)** The variability of the dynamic segregating process was negatively related to the avolition score in the default-mode network (r=-0.317, p=0.025) and control systems (r=-0.356, p=0.011). **e)** The alogia score are positively correlated with the variability of the dynamic segregating process in the dorsal attention system (r=0.284, p=0.046). Significant correlation results are similar for 500 regions and 200 regions.

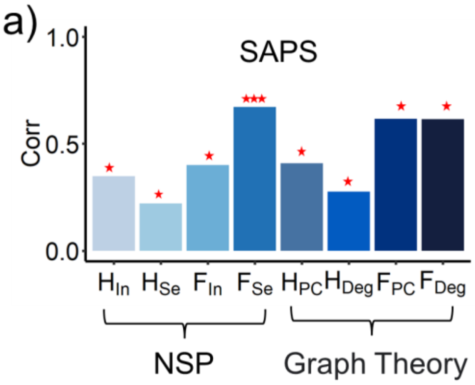

Supplementary Figure 7. Prediction of SANS and SAPS scores. a) Eight brain network measures integration strength H_{In} , segregation strength H_{Se} , integration variability F_{In} , segregation variability F_{Se} , degree strength H_{Deg} , participation coefficient strength H_{PC} , degree variability F_{Deg} and participation coefficient variability F_{PC} were used to predict the SAPS and **b)** SANS scores. The bar charts are correlations between real and predicted scores. ***p<0.001, **p<0.01, *p<0.05. In the machine learning model, NSP measures outperforms the classical graph theory measures in predicting the SAPS and SANS scores.

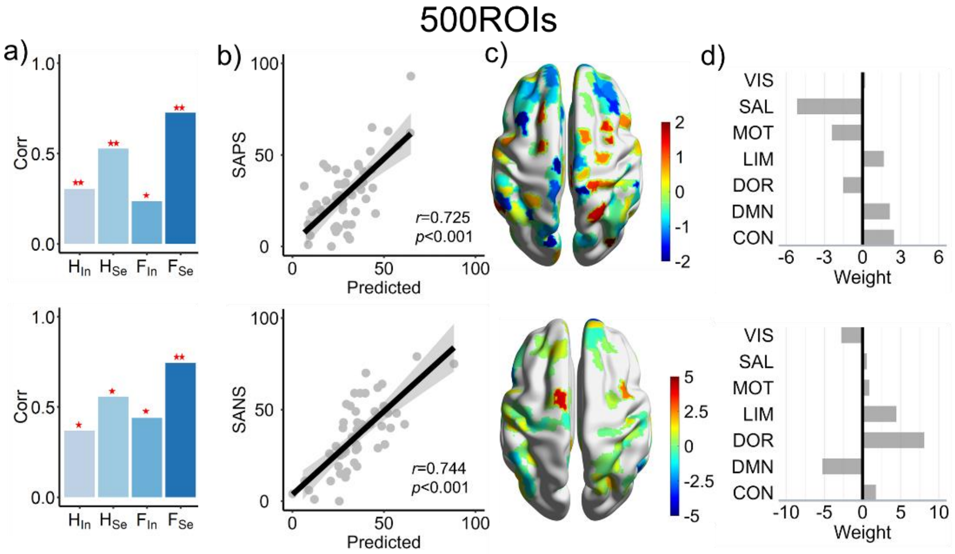

Supplementary Figure 8. Predicting SANS/SAPS score in brain network with 500 regions. a) Using a machine learning approach, four brain network measures integration strength H_{In}, segregation strength H_{Se}, integration variability F_{In}, segregation variability F_{Se} were used to predict the SAPS (upper panel) and SANS (lower panel) scores. Segregation variability F_{Se} is the best measure in predicting the SAPS score (r=0.725, p<0.001) and SANS score (r=0.744, p<0.001). **b)** The correlation between SAPS/SANS scores and predicted scores for segregation variability F_{Se}. **c)** The weights of regions in the best prediction were mapped to the brain surface. **d)** In the SAPS prediction model, the salient attention system had high positive weights, and the control and DMN system had high negative weight. In the SANS prediction model, the dorsal attention system had a high positive weight, and the DMN system had a high negative weight. The DMN system has opposite contributions to the predictions of SANS and SAPS scores. These results are similar to those from the parcellation of 200 regions.

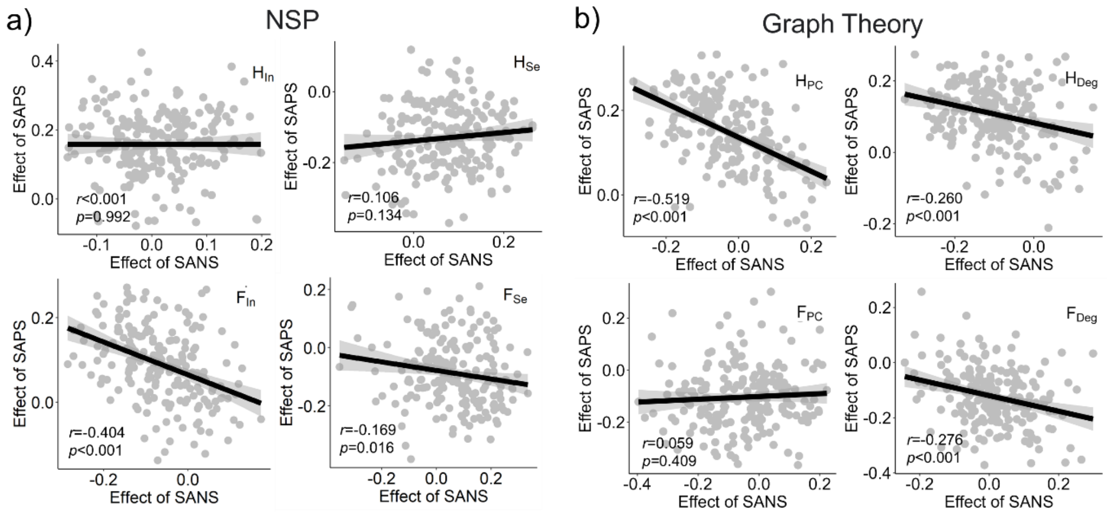

Supplementary Figure 9. Effects of schizophrenia symptoms on brain networks. The correlation between the effect of SANS and the effect of SAPS for **a)** NSP and **b)** graph theory measures. The opposite effects of SAPS and SANS on regions were found for both graph theory and NSP measures. For the NSP method, the effect of SAPS and the effect of SANS on regions are negatively correlated for dynamic integration variability F_{In} , segregation variability F_{Se} . For graph theory measures, the effect of SAPS and the effect of SANS on regions are negatively correlated for dynamic degree variability F_{Deg} , degree strength H_{Deg} and participation coefficient strength H_{PC} .

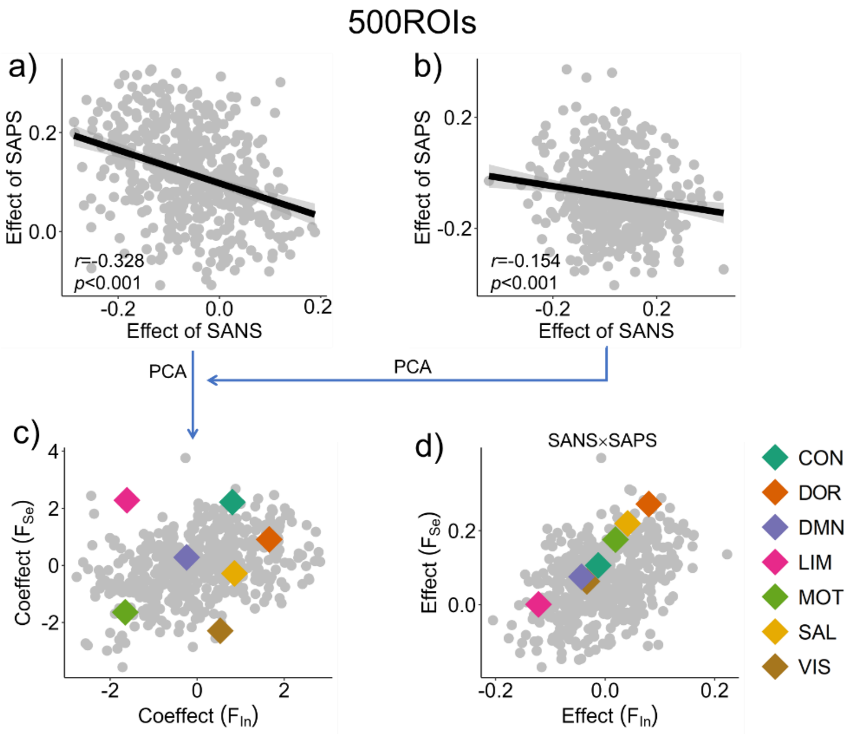

Supplementary Figure 10. Effects of schizophrenia symptoms on brain networks with 500 regions. a) The effect of SAPS and the effect of SANS on regions are negatively correlated for dynamic variabilities of segregation variability F_{Se} and **b)** integration variability F_{In}. **c)** Co-effects of SANS/SAPS on integration variability F_{In} and segregation variability F_{Se}. The co-effects were obtained from **a)** and **b)** using PCA. The averaged co-effect of SANS and SAPS on regions within each system was marked. SAPS predominantly affects the dynamic segregating and integrating process in motor system. SANS dominantly affects the integration process of the dorsal attention system and the segregation process of the limbic system. **d)** SANS and SAPS interactively affect both segregating and integrating processes in attention systems. These results are similar to those from the parcellation of 200 regions.

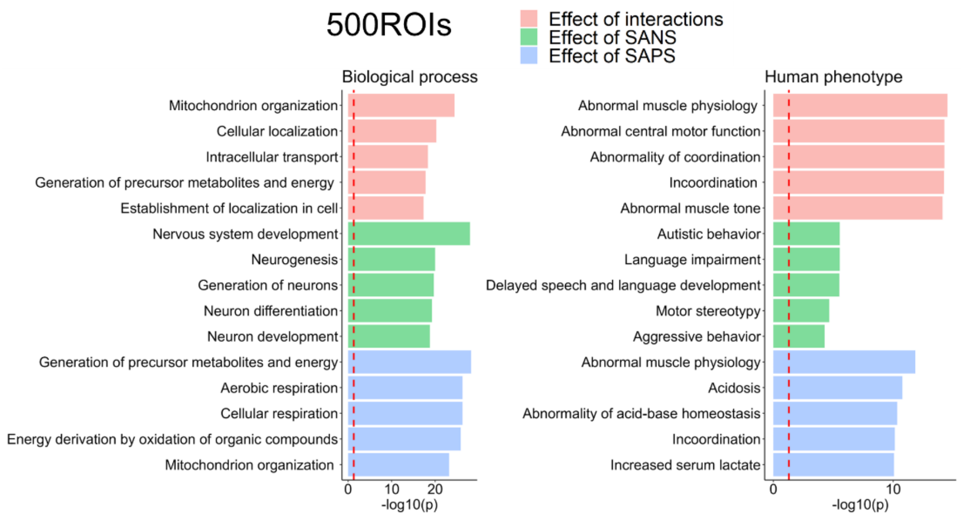

Supplementary Figure 11. GO enrichment results for biological processes and human phenotypes using ToppGene. We used these significant genes to perform GO annotation analysis using the ToppGene Suite. The overall effect of SANS is mainly related to biological nervous system development, and the corresponding human phenotypes include abnormal autistic, language and aggressive behavior. The overall effect of SAPS is related to the generation of precursor metabolites and energy and aerobic respiration. The corresponding human phenotypes include abnormal muscle physiology and acidosis. SANS×SAPS interaction is mainly related to biological mitochondrion organization and cellular localization, and the corresponding human phenotypes are abnormal muscle physiology and abnormal central motor function. These results are similar to those from the parcellation of 200 regions.

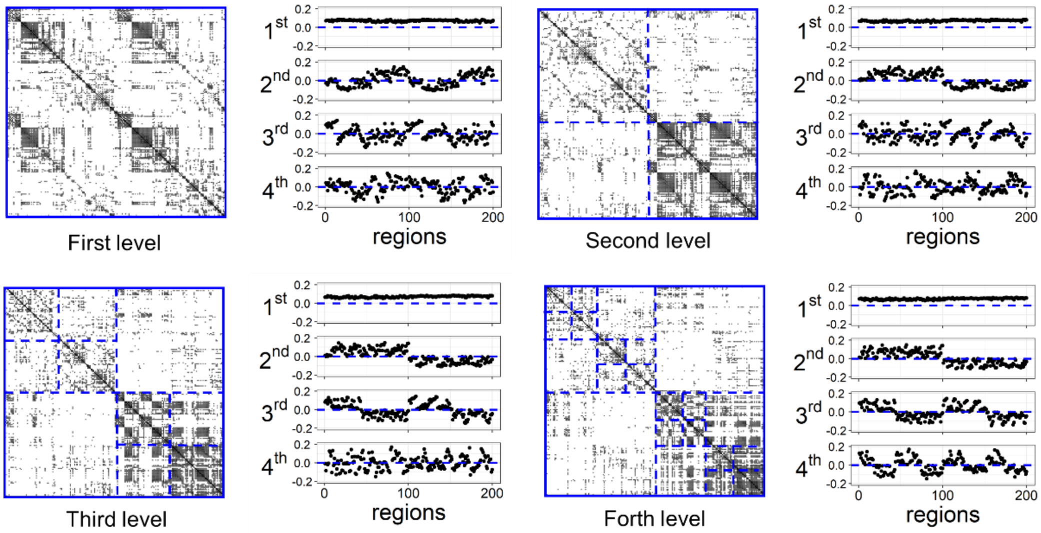

Supplementary Figure 12. The partition of the FC network into hierarchical modules in the first four levels. The blue dashed lines represent the boundaries of hierarchical modules suggested by the NSP method. Note that the FC network was binarized for clearer illustration, but the NSP method was performed at weighted networks. A hierarchical modular partition in the first four functional modes was also provided, where modules in each level were detected according to the positivity or negativity of eigenvector components. Note that after each partitioning step, the regions were reordered, and the order within modules remained random.

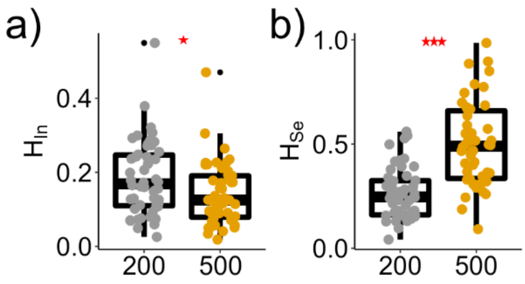

Supplementary Figure 13. Higher segregation in larger-size brain functional network. a) Compared to the 200 regions healthy control (HC) group, the 500 regions HC group had decreased integration strength ($t(98)=-16.649$, $p<0.001$) and **b)** increased segregation strength ($t(98)=20.136$, $p<0.001$).

Supplementary Table 1. Correlations between eight measures, integration strength H_{In} , segregation strength H_{Se} , integration variability F_{In} , segregation variability F_{Se} , degree strength H_{Deg} , participation coefficient strength H_{PC} , degree variability F_{Deg} , participation coefficient variability F_{PC} and SANS/SAPS scores in the whole-brain network.

corr (*p*-value)	H_{In}	H_{Se}	F_{In}	F_{Se}	H_{PC}	H_{Deg}	F_{PC}	F_{Deg}
SANS	0.066 (0.649)	0.058 (0.690)	-0.044 (0.761)	0.074 (0.610)	-0.011 (0.937)	-0.082 (0.574)	-0.030 (0.839)	-0.136 (0.348)
SAPS	0.160 (0.268)	-0.114 (0.429)	0.058 (0.690)	-0.105 (0.470)	0.228 (0.111)	0.109 (0.451)	-0.133 (0.358)	-0.141 (0.330)

Supplementary Table 2. Thirty-two schizophrenia-related genes with damaging ultra-rare mutations have been found in previous articles, and our data included 27 of them (first 27 lines in the table). We identified 12 genes associated with SANS and SAPS, and 9 genes were related to SANS×SAPS interaction.

**Gene_name**	**Schizophrenia symptoms**
GRIN2A	SANS×SAPS
HERC1	
AKAP11	
DNM3	
ASH1L	
PREP	
MAGI2	
DAGLA	SANS×SAPS, SAPS
SV2A	
SETD1A	SANS
CACNA1G	
ANKRD12	
CUL1	
XPO7	
TRIO	
SP4	
GRIA3	
RB1CC1	
HCN4	
ZNF136	
SRRM2	
NR3C2	
ZMYM2	
FAM120A	
STAG1	
HIST1H1E	
EIF2S3	
FAM178A	
KDM6B	
MAGEC1	
OR4P4	
SLC22A11	

Supplementary Table 3. Correlations between global signal fluctuation and network measures: integration strength H_{In} , segregation strength H_{Se} , integration variability F_{In} , segregation variability F_{Se} in the whole-brain (ALL) and systems. Significant correlations were marked in red colors.

corr (*p*-value)	ALL	VIS	MOT	DOR	SAL	LIM	CON	DMN
H_{In}	0.689 (0.001)	0.485 (0.001)	0.430 (0.002)	0.512 (0.001)	0.469 (0.001)	0.447 (0.001)	0.539 (0.001)	0.543 (0.001)
H_{Se}	-0.593 (0.001)	-0.558 (0.001)	-0.441 (0.001)	-0.533 (0.001)	-0.502 (0.001)	-0.539 (0.001)	-0.582 (0.001)	-0.556 (0.001)
F_{In}	0.526 (0.001)	0.391 (0.005)	0.247 (0.084)	0.309 (0.029)	0.265 (0.063)	0.408 (0.003)	0.349 (0.013)	0.355 (0.011)
F_{Se}	-0.522 (0.001)	-0.317 (0.025)	-0.404 (0.004)	-0.380 (0.006)	-0.300 (0.034)	-0.071 (0.625)	-0.234 (0.102)	-0.091 (0.530)
