# Supplementary material for: Opposite effects of positive and negative symptoms on resting-state brain networks in schizophrenia
Source: Commun Biol. 2023 Mar 17;6:279. doi: 10.1038/s42003-023-04637-0 (PMC10023794; doi:10.1038/s42003-023-04637-0)
Supplement: Supplementary file 3 — Description of Additional Supplementary Files [file 42003_2023_4637_MOESM3_ESM.pdf]

Description of Additional Supplementary Files

File name: Supplementary Data 1

Description: Pearson correlation coefficients and corresponding correlation p-values were evaluated between brain network measures and symptom scores.

File name: Supplementary Data 2

Description: P-value of brain network measures in multivariable regression models.

File name: Supplementary Data 3

Description: The Scale for the Assessment of Negative Symptoms (SANS).

File name: Supplementary Data 4

Description: The Scale for the Assessment of Positive Symptoms (SAPS).
