# Supplementary material for: Opposite effects of positive and negative symptoms on resting-state brain networks in schizophrenia
Source: Commun Biol. 2023 Mar 17;6:279. doi: 10.1038/s42003-023-04637-0 (PMC10023794; doi:10.1038/s42003-023-04637-0)
Supplement: Supplementary file 8 — Reporting Summary [file 42003_2023_4637_MOESM8_ESM.pdf]

Corresponding author(s): Rong Wang
Last updated by author(s): Feb 15, 2023

Reporting Summary

Nature Portfolio wishes to improve the reproducibility of the work that we publish. This form provides structure for consistency and transparency in reporting. For further information on Nature Portfolio policies, see our Editorial Policies and the Editorial Policy Checklist.

Statistics

For all statistical analyses, confirm that the following items are present in the figure legend, table legend, main text, or Methods section.

n/a	Confirmed	
☐	☒	The exact sample size (*n*) for each experimental group/condition, given as a discrete number and unit of measurement
☒	☐	A statement on whether measurements were taken from distinct samples or whether the same sample was measured repeatedly
☐	☒	The statistical test(s) used AND whether they are one- or two-sided<br>*Only common tests should be described solely by name; describe more complex techniques in the Methods section.*
☐	☒	A description of all covariates tested
☐	☒	A description of any assumptions or corrections, such as tests of normality and adjustment for multiple comparisons
☐	☒	A full description of the statistical parameters including central tendency (e.g. means) or other basic estimates (e.g. regression coefficient) AND variation (e.g. standard deviation) or associated estimates of uncertainty (e.g. confidence intervals)
☐	☒	For null hypothesis testing, the test statistic (e.g. *F*, *t*, *r*) with confidence intervals, effect sizes, degrees of freedom and *P* value noted<br>*Give P values as exact values whenever suitable.*
☒	☐	For Bayesian analysis, information on the choice of priors and Markov chain Monte Carlo settings
☐	☒	For hierarchical and complex designs, identification of the appropriate level for tests and full reporting of outcomes
☐	☒	Estimates of effect sizes (e.g. Cohen's *d*, Pearson's *r*), indicating how they were calculated

Our web collection on statistics for biologists contains articles on many of the points above.

Software and code

Policy information about availability of computer code

Data collection	The dataset was extracted from the UCLA Consortium for Neuropsychiatric Phenomics LA5c Study(https://openneuro.org/datasets/ds000030/versions/1.0.0).
Data analysis	Matlab(2020a) was used to do the statistical and classification analysis. Python(3.9.6) was used to build the machine learning models. RStudio(4.1.0) was used to generate figure.

For manuscripts utilizing custom algorithms or software that are central to the research but not yet described in published literature, software must be made available to editors and reviewers. We strongly encourage code deposition in a community repository (e.g. GitHub). See the Nature Portfolio guidelines for submitting code & software for further information.

Data

Policy information about availability of data

All manuscripts must include a data availability statement. This statement should provide the following information, where applicable:

- Accession codes, unique identifiers, or web links for publicly available datasets
- A description of any restrictions on data availability
- For clinical datasets or third party data, please ensure that the statement adheres to our policy

The dataset was extracted from the UCLA Consortium for Neuropsychiatric Phenomics LA5c Study(https://openneuro.org/datasets/ds000030/versions/1.0.0).

Human research participants

Policy information about studies involving human research participants and Sex and Gender in Research.

Reporting on sex and gender	Fifty schizophrenia patients (female: 12, male: 38, age: 36.46 ± 8.88 years old) and 50 healthy controls (female: 12, male: 38, age: 34.84 ± 9.03 years old) were included. There was no significant difference in sex and age (two-sample t test, t(98)=0.905, p=0.354).
Population characteristics	To be included individuals had to be either 'White, Not of Hispanic or Latino Origin' or 'Hispanic or Latino, of Any Race' following NIH designations of racial and ethnic minority groups, and have completed at least 8 years of education (other racial and ethnic minority groups were excluded because this was thought to increase risk of confounding planned genetic studies). For participants who spoke both English and Spanish, language for testing was determined by a verbal fluency test. Participants were screened for neurological disease, history of head injury with loss of consciousness or cognitive sequelae, use of psychoactive medications, substance dependence within past 6 months, history of major mental illness or ADHD, and current mood or anxiety disorder. Self-reported history of psychopathology was verified with the SCID-IV (First, Spitzer, Gibbon, & Williams, 1995). Urinalysis was used to screen for drugs of abuse (cannabis, amphetamine, opioids, cocaine, benzodiazepines) on the day of testing and excluded if results were positive.
Recruitment	Subjects were recruited by community advertisement and through outreach to local clinics and online portals. How participants were recruited had a small and negligible effect on the results.
Ethics oversight	All studies were conducted in accordance with principles for human experimentation as defined in the Declaration of Helsinki and International Conference on Harmonization Good Clinical Practice guidelines. All participants gave written informed consent according to the procedures approved by the University of California Los Angeles Institutional Review Board.

Note that full information on the approval of the study protocol must also be provided in the manuscript.

Field-specific reporting

Please select the one below that is the best fit for your research. If you are not sure, read the appropriate sections before making your selection.

☒ Life sciences ☐ Behavioural & social sciences ☐ Ecological, evolutionary & environmental sciences

For a reference copy of the document with all sections, see nature.com/documents/nr-reporting-summary-flat.pdf

Life sciences study design

All studies must disclose on these points even when the disclosure is negative.

Sample size	We select 50 schizophrenia patients and 50 healthy controls from UCLA Consortium for Neuropsychiatric Phenomics LA5c Study dataset .
Data exclusions	No data was excluded.
Replication	The results were verified using the co-registration software for several times, which showed consistency.
Randomization	The mean framewise displacement (FD) was 0.160 ± 0.159 mm for the healthy control group and 0.267 ± 0.215 mm for the schizophrenia group. The difference in FD between the two groups was significant (two-sample t test, t(98)=2.779, p=0.004). Thus, an analysis of covariance (ANCOVA) was carried out for the group comparison.
Blinding	Subjects were grouped based on whether they diagnosed as schizophrenia or not. The investigators were blinded to group allocation during data collection and/or analysis.

Reporting for specific materials, systems and methods

We require information from authors about some types of materials, experimental systems and methods used in many studies. Here, indicate whether each material, system or method listed is relevant to your study. If you are not sure if a list item applies to your research, read the appropriate section before selecting a response.

Materials & experimental systems

n/a	Involved in the study
☒	☐ Antibodies
☒	☐ Eukaryotic cell lines
☒	☐ Palaeontology and archaeology
☒	☐ Animals and other organisms
☒	☐ Clinical data
☒	☐ Dual use research of concern

Methods

n/a	Involved in the study
☒	☐ ChIP-seq
☒	☐ Flow cytometry
☐	☒ MRI-based neuroimaging

Magnetic resonance imaging

Experimental design

Design type

resting-state

Design specifications

In the Resting scan, participants were asked to remain relaxed and keep their eyes open for five minutes. They were not presented any stimuli or asked to respond during the scan. A subset of the larger healthy sample and patient sample took part in two separate fMRI sessions, which each included one hour of behavioral testing and a one-hour scan on the same day.

Behavioral performance measures

"For the following part, you do not need to do anything other than keep your eyes open and remain relaxed. It is still important that you remain still and do not fall asleep, but you don't need to respond to anything. Any questions?"

Acquisition

Imaging type(s)

functional

Field strength

3T

Sequence & imaging parameters

maging data were acquired on a 3T Siemens Trio scanner. Functional MRI data were collected with a T2*-weighted echoplanar imaging (EPI) sequence with parameters: slice thickness = 4mm, 34 slices, TR=2s, TE=30ms, flip angle=90°, matrix=64 × 64, FOV=192mm. A T1-weighted high-resolution anatomical scan (MPRAGE) were collected with the following parameter: slice thickness = 1mm, 176 slices, TR=1.9s, TE=2.26ms, matrix=256 × 256, FOV=250mm. Diffusion weighted imaging data were collected with parameters: slice thickness = 2mm, 64 directions, TR/TE=9000/93ms, flip angle=90°, matrix=96 × 96, axial slices, b=1000s/mm2.

Area of acquisition

Whole brain

Diffusion MRI ☐ Used ☒ Not used

Preprocessing

Preprocessing software

AFNI (http://afni.nimh.nih.gov/afni/) and FSL (http://www.fmrib.ox.ac.uk/fsl/) were used to preprocess the resting-state fMRI data.

Normalization

All T1-weighted images were skull-stripped20 [AFNI 3dSkullStrip], corrected for intensity inhomogeneity due to B1 variations21 [ANTS N4], and normalized to MNI-152 2 mm template space.

Normalization template

The procedure included 1) slice-timing correction to the median slice; 2) motion correction; 3) segmenting the anatomical image; 4) Montreal Neurological Institute (MNI) normalization; 5) spatial smoothing using a Gaussian kernel with a 6-mm full width at half maximum (FWHM); 6) bandpass filtering (0.01-0.1 Hz); and 7) elimination of 6 rigid body motion correction parameters and the signal from the white matter and a ventricular region of interest using linear regression. The mean framewise displacement (FD) was 0.160±0.159 mm for the healthy control group and 0.267±0.215 mm for the schizophrenia group. The difference in FD between the two groups was significant (two-sample t test, t(98)=2.779, p=0.004). Thus, an analysis of covariance (ANCOVA) was carried out for the group comparison. Since the global whole-brain signal was related to brain network integration and segregation (Table S1) and may contain the clinical information of schizophrenia symptoms, it was not removed from our analysis.

Noise and artifact removal

Several sources of nuisance covariates were eliminated using linear regression: 1) 6 rigid body motion correction parameters and 2) the signal from the white matter and from a ventricular region of interest.

Volume censoring

Framewise displacement (FD) and DVARS. FD is calculated from derivatives of the sixrigid-body realignment parameters estimated during standard volume realignment,and is a compressed single six index of the realignment parameters. DVARS is the root mean squared (RMS)change in BOLD signal from volume to volume(referring to temporal derivative of timecourses and VARS referring to RMS varianceover voxels). DVARS is calculated by firstdifferentiating the volumetric time series andthen calculating the RMS signal change overthe whole brain. This measure indexes thechange rate of BOLD signal across the entirebrain at each frame of data or, in other words,how much the intensity of a brain imagechanges relative to the previous time point.

Statistical modeling & inference

Model type and settings	Multivariate and predictive.
Effect(s) tested	There was no task conditions, therefore no effects were tested.

Specify type of analysis: ☐ Whole brain ☐ ROI-based ☒ Both

Anatomical location(s)	The brain was parcellated into N=200 regions of interest (ROIs) using the Schaefer atlas and compare with the brain parcellation of 500 regions.
Statistic type for inference (See Eklund et al. 2016)	Whole brain and subsystem voxel wise analysis.
Correction	FDR, permutation

Models & analysis

n/a	Involved in the study
☐	☒ Functional and/or effective connectivity
☐	☒ Graph analysis
☐	☒ Multivariate modeling or predictive analysis

Functional and/or effective connectivity

The brain was parcellated into N=200 regions of interest (ROIs) using the Schaefer atlas. The blood-oxygen level-dependent (BOLD) signals of voxels within each region were averaged to obtain the regional fMRI time series, and the Pearson correlation coefficient was used to estimate the FC between regions. The BOLD signals were divided into pieces using the sliding window method, and temporal-dynamic FC was calculated in each window. As suggested by Leonardi et al. 39, we chose a window width of 60 s (30 points) and a sliding step of 2 s (1 point), and there were 132 windows. Meanwhile, group-stable, individual static FC networks were also constructed, which were used to address the limitation of shorter fMRI series lengths resulting in stronger network segregation 32 (see fMRI length calibration). For the group-stable FC, the fMRI time series for all participants in each group were concentrated, and the FC was computed on a long enough time scale. Individual static FC networks were constructed using the whole fMRI time series in each participant. In all FC networks, negative connectivity was set to zero, and the diagonal elements were kept at one.

Graph analysis

We have compared the graph theory measures (participation coefficient and degree) with our NSP measures.

Multivariate modeling and predictive analysis

Machine learning prediction model: The scikit-learn toolbox was used to construct a machine learning prediction model. First, we used the function linear_model.Linear Regression to build linear predictive models. The independent variables were regional measures (i.e.,Hin, Hse, Fin, Fse, PC and Deg), and the dependent variables were the SANS or SAPS scores. Second, leave-one-out cross validation (LOO-CV) was applied with the function cross_val_predict. In each iteration of LOO-CV, one sample was selected as the test set, and the remaining samples were selected as the training set. This process was repeated until every participant had been selected as a test set once. Then, we used the correlation between the real clinical score and the predicted score to evaluate the prediction accuracy, and the statistical comparison was performed by permuting the ranks of clinical scores (10000 times). In the prediction model, the functions f_regression and SelectKBest were used to select features. The f_regression function calculated the correlations between regional measures and clinical scores and sorted the regions according to their F values. Then, the first K features were selected and fed into the prediction model. Here, we varied K from 1 to N and chose the best K, defined as the value at which the model had the best predictive performance. The input features were normalized such that the weights of regions were comparable.
Effects of SANS and SAPS on the brain: To extract the effects of positive and negative symptoms, as well as their interaction effect on brain FC networks, we built a multiple regression model: H~SANS+SAPS +SANS×SAPS+age+sex+FD. Here, H is the brain measure for each region, i.e.,Hin, Hse, Fin and Fse. The regression coefficients of SANS and SAPS reflect the effects of negative and positive symptoms on the brain, and the coefficient of SANS×SAPS indicates the interaction effect.
